# Subtyping Internet Gaming Disorder Based on Interindividual Deviation of Functional Connectivity: A Network Contingency Analysis

**DOI:** 10.1111/adb.70184

**Published:** 2026-09-10

**Authors:** Yichen Guo, Xinyu Wang, Wenjing Li, Guohao Lu, Yang Liu, Sha Zhou, Kaixin Li, Mengzhu Wang, Yan Lang, Hongwei Zheng, Yong Zhang, Weijian Wang

**Affiliations:** ^1^ Department of Magnetic Resonance Imaging First Affiliated Hospital of Zhengzhou University Zhengzhou Henan China; ^2^ MR Research Collaboration Siemens Healthineers Ltd Beijing China; ^3^ Department of Psychiatry First Affiliated Hospital of Zhengzhou University Zhengzhou Henan China

**Keywords:** functional connectivity, interindividual deviation, internet gaming disorder functional magnetic resonance imaging, subtype

## Abstract

Internet gaming disorder (IGD) is a behavioural addiction characterized by substantial clinical heterogeneity. The present study aimed to investigate this heterogeneity by analysing the deviation patterns of interindividual brain functional connectivity. Resting‐state functional magnetic resonance imaging (fMRI) data from 87 patients with IGD and 56 healthy controls (HCs) were included. First, functional connectivity matrices were constructed, and the interindividual deviation of functional connectivity (IDFC) of each patient with IGD relative to the HC group was quantified at the network level. K‐means clustering was then applied to identify IGD subtypes. Edge‐wise functional connectivity analysis with network contingency analysis (NCA) was used to characterize subtype‐specific patterns of altered connections. Finally, support vector regression (SVR) based on network‐level IDFC features was used to predict Internet Addiction Test (IAT) scores in the overall IGD cohort. Two distinct IGD subtypes were identified based on IDFC patterns. Compared with HCs, Subgroup 1 showed a widespread hypoconnectivity pattern, whereas Subgroup 2 showed a relative hyperconnectivity pattern. Network‐level IDFC features also predicted IAT scores in the overall IGD cohort using SVR (*r* = 0.391, one‐tailed permutation *p* = 0.038). Patients with IGD exhibit substantial interindividual variability in brain functional connectivity. Subtyping IGD based on these connectivity deviation patterns may improve the understanding of its neurobiological heterogeneity and facilitate the development of more precise individualized interventions.

## Graphical abstract

Internet gaming disorder (IGD) shows substantial clinical heterogeneity. Using resting‐state fMRI data from 87 IGD patients and 56 healthy controls, we identified two distinct IGD subtypes based on interindividual deviation of functional connectivity (IDFC). Subgroup 1 exhibited widespread hypoconnectivity and higher addiction severity, whereas Subgroup 2 showed relative hyperconnectivity. Network‐level IDFC features predicted Internet Addiction Test scores. These findings suggest that IGD comprises neurobiologically distinct subtypes, supporting the need for stratified approaches in IGD research and personalized interventions.

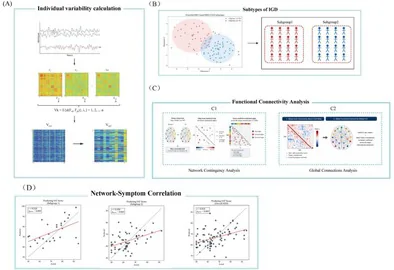

## 1 Introduction

Internet gaming disorder (IGD) is the most prevalent subtype of internet addiction disorder (IAD) accounting for approximately 57.5% of all IAD cases [1, 2]. IGD is characterized by an uncontrollable urge to engage in online gaming, resulting in significant social, occupational, financial and behavioural impairments [3]. Increasing evidence suggests that IGD exhibits substantial clinical heterogeneity, with previous studies identifying distinct subgroups based on psychological traits and comorbid symptoms [4]. However, the neurobiological basis underlying such heterogeneity remains poorly understood.

Neuroimaging studies have reported widespread structural and functional brain abnormalities in individuals with IGD, particularly in regions associated with executive control, salience processing, reward and affective regulation. Nevertheless, findings across studies remain inconsistent. For example, both increased and decreased functional connectivity patterns have been reported in similar brain regions in IGD populations [5, 6, 7]. Likewise, structural imaging studies have shown contradictory findings regarding grey matter alterations [8, 9]. One possible explanation for these inconsistencies is that conventional group‐level analyses rely on population averaging approaches, which may obscure highly individualized neural characteristics within heterogeneous patient populations [10, 11].

Recently, individualized neuroimaging frameworks have shown increasing value in characterizing heterogeneous psychiatric disorders [12, 13, 14]. Rather than focusing solely on group‐level differences, these approaches quantify deviations of individual brain patterns relative to healthy populations, thereby providing greater sensitivity for detecting subject‐specific neural abnormalities. In the context of IGD, previous studies have demonstrated the feasibility of identifying neurobiologically distinct subgroups using resting‐state functional connectivity (FC) patterns [15]. However, most existing studies classify IGD subgroups based only on intrinsic patient characteristics while neglecting interindividual deviations relative to healthy controls (HCs). Consequently, approaches based on interindividual deviation may provide a more effective framework for uncovering heterogeneous neural patterns in IGD.

The human brain is organized into multiple large‐scale intrinsic connectivity networks (ICNs), including the default mode network (DMN), executive control network (ECN), salience network (SN) and sensorimotor‐related networks [16, 17, 18, 19, 20, 21, 22]. Previous studies have consistently demonstrated abnormal functional interactions within and between these ICNs in IGD [18, 19, 20, 21, 22]. However, conventional FC analyses remain susceptible to a dilution effect caused by group averaging, potentially masking localized and individualized connectivity alterations. To address this limitation, the present study further incorporated network contingency analysis to identify systematic connectivity alterations between large‐scale network pairs [23].

In the present study, we investigated heterogeneity in IGD using an interindividual deviation framework based on resting‐state functional connectivity. First, individualized FC matrices were constructed for each participant, and IDFC relative to the HC group was quantified at the network level. Subsequently, clustering analysis based on IDFC features was performed to identify potential neurobiological subgroups of IGD. Edge‐wise functional connectivity analysis and NCA were then used to characterize subtype‐specific patterns of large‐scale network reorganization. Finally, SVR was applied to evaluate whether network‐level IDFC features could predict IAT scores in the overall IGD cohort. We hypothesized that (1) individuals with IGD could be subdivided into distinct subgroups based on network‐level IDFC features; (2) different IGD subgroups would exhibit distinct alterations in large‐scale intrinsic connectivity networks; and (3) network‐level IDFC features would be associated with addiction severity.

## 2 Materials and Methods

### 2.1 Participants

A total of 143 participants, including 87 individuals with IGD and 56 HCs, were recruited from the First Affiliated Hospital of Zhengzhou University. All participants were right‐handed. Demographically, the IGD group comprised 71 males and 16 females, whereas the HC group included 41 males and 15 females. IGD diagnosis was established according to the Diagnostic and Statistical Manual of Mental Disorders, Fifth Edition (DSM‐5) and the Internet Addiction Test (IAT). Experienced psychiatrists evaluated all participants using DSM‐5 diagnostic criteria for IGD. Internet gaming addiction severity was assessed using the IAT, a 20‐item self‐report questionnaire evaluating compulsive internet use, psychological dependence, withdrawal symptoms, impaired time management, sleep disturbance and social or occupational dysfunction. Sleep quality was assessed using the Pittsburgh Sleep Quality Index (PSQI). The IAT, PSQI, HAMA, HAMD, Y‐BOCS and mania assessments were collected for the IGD cohort only; therefore, clinical‐scale comparisons involving these measures were restricted to comparisons between IGD subgroups rather than IGD‐HC comparisons. Participants were assigned to the IGD group if they met at least five DSM‐5 diagnostic criteria for IGD and had an IAT score ≥ 50 [24]. Individuals with IAT scores < 50 who did not meet DSM‐5 criteria were classified as HCs. All participants additionally underwent structured clinical interviews using the Mini‐International Neuropsychiatric Interview (M.I.N.I.) conducted by experienced psychiatrists. Exclusion criteria included (1) anxiety disorders, major depressive disorder, substance use disorders or attention‐deficit/hyperactivity disorder (ADHD); (2) intellectual disability; and (3) neurological disorders or history of brain injury. Participants were instructed to avoid medication and psychoactive substances on the day of MRI scanning. The study was approved by the Ethics Committee of the First Affiliated Hospital of Zhengzhou University (2022‐KY‐0438) and was conducted in accordance with the Declaration of Helsinki. Written informed consent was obtained from all participants prior to enrollment. Demographic and clinical information are summarized in Table 1.

**TABLE 1:** Demographic and clinical characteristics of participants.

	IGD (*N* = 87)	HC (*N* = 56)	t/χ^2^ value	*p* value
Sex, male/female, *n*	71/16	41/15	0.894	0.344
Age (years)	14.55 ± 1.86	15.57 ± 5.51	1.342	0.184
Education (years)	8.60 ± 1.98	9.07 ± 4.14	0.787	0.434
Duration of illness (years)	3.17 ± 2.13	—	—	—
IAT score	62.89 ± 9.27	—	—	—
PSQI scores	8.69 ± 7.30	—	—	—
HAMA scores	14.78 ± 10.32	—	—	—
HAMD scores	21.73 ± 13.65	—	—	—
Y‐B scores	10.61 ± 8.63	—	—	—

*Note:* These clinical scales were collected only in the IGD cohort; HC values were therefore not available, and scale‐based comparisons were limited to IGD subgroup analyses.

Abbreviations: HAMA, Hamilton Anxiety Scale; HAMD, Hamilton Depression Scale; HC, healthy control; IAT, Internet Addiction Test; IGD, internet gaming disorder; PSQI, Pittsburgh Sleep Quality Index; Y‐BOCS, Yale‐Brown Obsessive Compulsive Scale.

### 2.2 Data Preprocessing

MRI data were acquired using a 3T MAGNETOM Prisma MRI scanner (Siemens Healthineers, Forchheim, Germany) equipped with a 64‐channel head coil. During scanning, participants were instructed to remain awake with their eyes closed, stay relaxed and avoid deliberate cognitive activity. Foam padding and earplugs were used to minimize head motion and scanner noise. Resting‐state functional images were acquired using a gradient‐echo sequence. The parameters were set as follows: repetition time (TR) = 1000 ms, echo time (TE) = 30 ms, flip angle = 70°, field of view (FOV) = 220 × 220 mm^2^, voxel size = 2 × 2 × 2.2 mm^3^, 52 slices and 400 volumes. Image preprocessing was performed using DPARSF. After converting the data format from DICOM to NIFTI, we removed the initial five volumes to ensure signal equilibrium. Subsequently, slice timing correction and head motion realignment were executed on the remaining images. Any participant demonstrating head motion exceeding 2.5 mm in translation or 2.5° in rotation was excluded from further analysis. The functional images were then aligned to the standard EPI template for spatial normalization and resampled to 3 × 3 × 3 mm^3^. Spatial smoothing was performed using a 6‐mm full‐width at half‐maximum (FWHM) Gaussian kernel. Finally, to reduce the effects of physiological noise and residual head motion, nuisance covariates including Friston‐24 motion parameters, white matter signals and cerebrospinal fluid signals were regressed out [25, 26, 27]. Linear detrending was additionally applied.

### 2.3 Interindividual Deviation of Functional Connectivity

**FIGURE 1:**
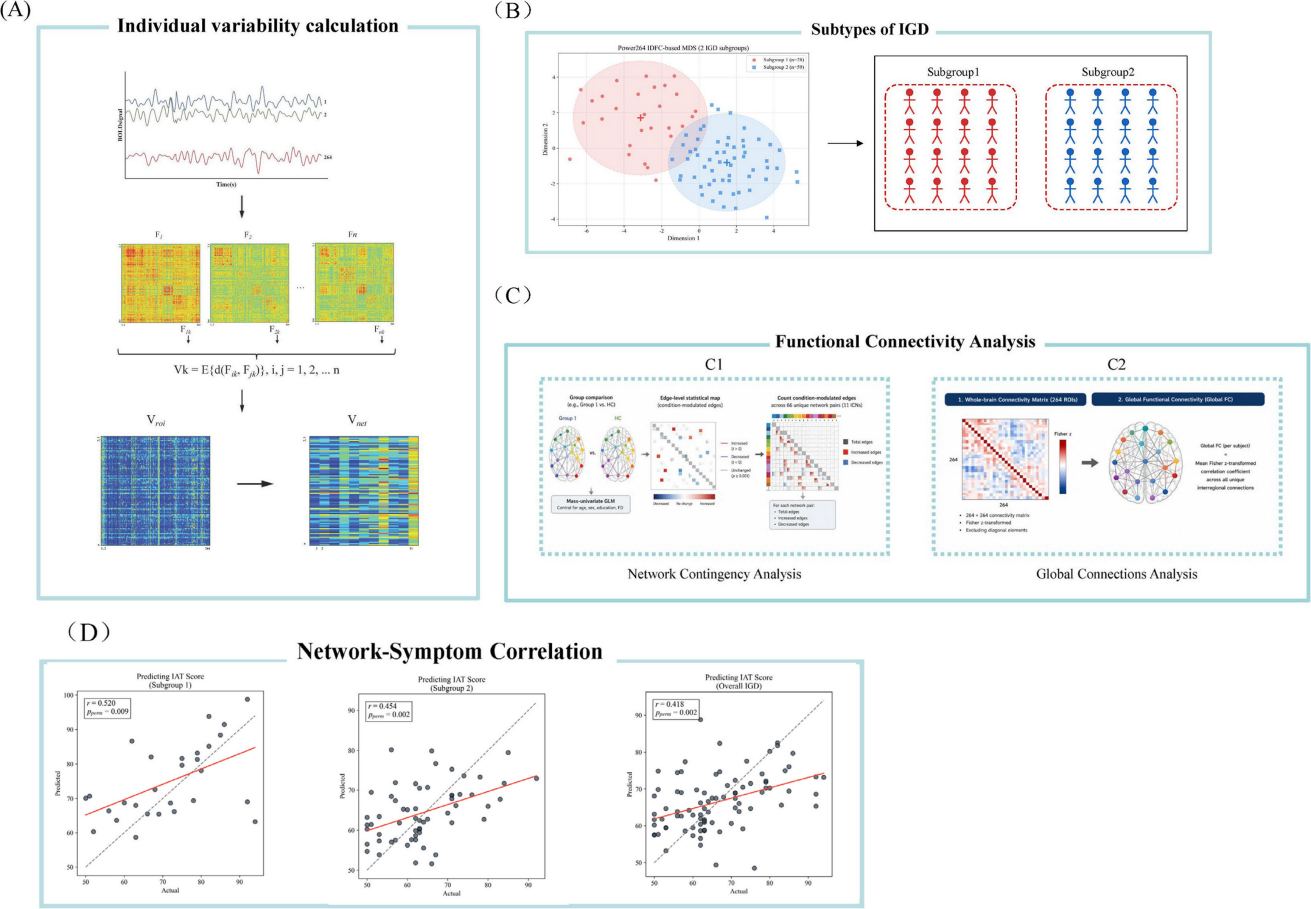
Study workflow. (A) Construction of individual deviation features from resting‐state functional connectivity. ROI time series were extracted from the Power 264‐region atlas and used to construct individual functional connectivity matrices. Interindividual deviation of functional connectivity (IDFC) was quantified using cosine distance relative to the HC reference group, yielding ROI‐level and network‐level IDFC features. *d* denotes cosine distance; Vroi and Vnet represent ROI‐ and network‐level IDFC matrices, respectively. (B) Identification of IGD subgroups based on network‐level IDFC features. *K*‐means clustering was applied to the residualized and standardized network‐level IDFC features, resulting in two IGD subgroups. (C) Functional connectivity‐based group‐level network analysis. Fisher *z*‐transformed functional connectivity matrices were analysed using edge‐wise general linear models, and significant edges were summarized at the network‐pair level using network contingency analysis (NCA). Global functional connectivity was also calculated as the mean whole‐brain functional connectivity and compared among HCs, Subgroup 1 and Subgroup 2 using ANOVA followed by Bonferroni‐corrected post hoc tests. (D) Machine‐learning prediction of Internet gaming addiction severity. Network‐level IDFC features were used to predict Internet Addiction Test (IAT) scores in the overall IGD cohort using LinearSVR with leave‐one‐out cross‐validation (LOOCV). Prediction performance was evaluated by comparing predicted and observed IAT scores, and statistical significance was assessed using permutation testing. Averaged feature weights were used to summarize the contribution of each network‐level feature.

In this study, time series were extracted from 264 regions of interest (ROIs) defined by the Power atlas and grouped into 11 large‐scale functional networks, including sensory‐motor network (SMN), cingulo‐opercular network (CO), auditory network (AN), default mode network (DMN), visual network (VN), fronto‐parietal network (FPN), salience network (SAN), subcortical network (SUB), ventral attention network (VAN) and dorsal attention network (DAN). ROIs that could not be clearly assigned to any of these networks were classified as the uncertain network (UND) [28, 29]. For each subject, Pearson correlation coefficient was calculated between the time series of each ROI to measure functional connectivity (FC) between brain regions, resulting in a 264 × 264 functional connectivity matrix. Due to the current lack of consensus on the physiological interpretation of negative correlations, all negative values in the matrix were set to zero in this study [16, 30]. For each IGD participant, interindividual deviation of functional connectivity (IDFC) was quantified relative to the HC group. Specifically, the FC profile of each ROI was defined as the corresponding row vector of the FC matrix. Cosine distances between the FC profile of each IGD participant and all HC participants were calculated, and the mean cosine distance was defined as the IDFC value for that ROI. Region‐level IDFC values were subsequently averaged within each functional network to generate network‐level IDFC features (as shown in Figure 1A).

### 2.4 Clustering Analysis Based on IDFC

After extracting network‐level IDFC features across the 11 network labels, potential effects of age, sex, mean framewise displacement (mean FD) and years of education were regressed out using multiple linear regression. The residuals were standardized using *Z* scores and entered into subsequent machine‐learning analyses. *K*‐means clustering was applied to the IGD cohort to identify neurobiological clusters with similar deviation patterns (Figure 1B). The number of clusters (*k*) was explored from 2 to 7. Clustering performance was evaluated using the Calinski–Harabasz index, Davies–Bouldin index, silhouette score and within‐cluster sum of squares. To assess clustering robustness, 1000 iterations of 80% subsampling were performed. In each iteration, covariate regression, standardization and *k*‐means clustering were repeated, and agreement with the primary clustering solution was quantified using the adjusted Rand index (ARI) and normalized mutual information (NMI). Additional sensitivity analyses were performed using Ward hierarchical clustering, Gaussian mixture modelling, signed‐FC features and global‐FC‐regressed features. Subgroup differences in available clinical scale scores, including IAT, HAMD, HAMA, Y‐BOCS and mania scores, were evaluated using Welch two‐sample *t* tests and reported in the Supporting Information.

### 2.5 Functional Connectivity‐Based Network Analysis

To characterize large‐scale network alterations across IGD subgroups and HCs, FC analyses were performed at both edge and network levels. Pearson correlation matrices were Fisher *z*‐transformed before statistical analysis. A general linear model (GLM) was applied to each of the 34 716 unique functional connections while controlling for age, sex, years of education, mean FD and mean FD squared. Consistent with previous studies [23, 31], a primary threshold of *p* < 0.001 was applied to identify condition‐related edges. Significant edges were categorized as increased (*t* > 0) or decreased (*t* < 0) connectivity. For each within‐ and between‐network pair, the numbers of altered edges were quantified using NCA. Statistical significance was assessed using 10 000 Freedman–Lane permutations, and FDR correction was applied across network pairs. Directionality was summarized as (number of increased edges − number of decreased edges) / total number of altered edges.

### 2.6 Global Functional Connectivity Analysis

Global functional connectivity (Global FC) was calculated as the mean Fisher *z*‐transformed correlation coefficient across all unique interregional connections within the whole‐brain FC matrix. Between‐group differences in Global FC among Subgroup 1, Subgroup 2 and HCs were first assessed using one‐way ANOVA, followed by post hoc pairwise comparisons with Bonferroni correction. Corrected *p* < 0.05 was considered statistically significant (Figures 1C and S3).

### 2.7 Machine Learning‐Based Prediction Analysis

To investigate the relationship between network‐level features and the severity of Internet gaming addiction, prediction analysis was conducted in the overall IGD cohort (*n* = 87). The IAT/Young score was used as the dependent variable, and the 11 network‐level IDFC features were used as predictors. A linear support vector regression model with L2 regularization (LinearSVR) was applied [32]. Feature standardization was performed within each training fold and then applied to the corresponding test subject. Model performance was evaluated using leave‐one‐out cross‐validation (LOOCV), in which the model was trained on *n* − 1 subjects and tested on the remaining subject. Prediction accuracy was quantified by calculating the Pearson correlation coefficient (*r*) between predicted and observed IAT scores. Statistical significance was assessed using a one‐tailed nonparametric permutation test because the a priori hypothesis was that predicted and observed IAT scores would show a positive association. A total of 10 000 permutations were performed; in each permutation, IAT scores were randomly shuffled and the full LOOCV procedure was repeated. The permutation *p* value was calculated as (number of permuted correlations greater than or equal to the observed correlation + 1) / (number of permutations + 1). Feature weights were averaged across LOOCV folds to aid interpretation and are reported in the Supporting Information.

## 3 Results

### 3.1 Subtyping IGD Based on IDFC

**FIGURE 2:**
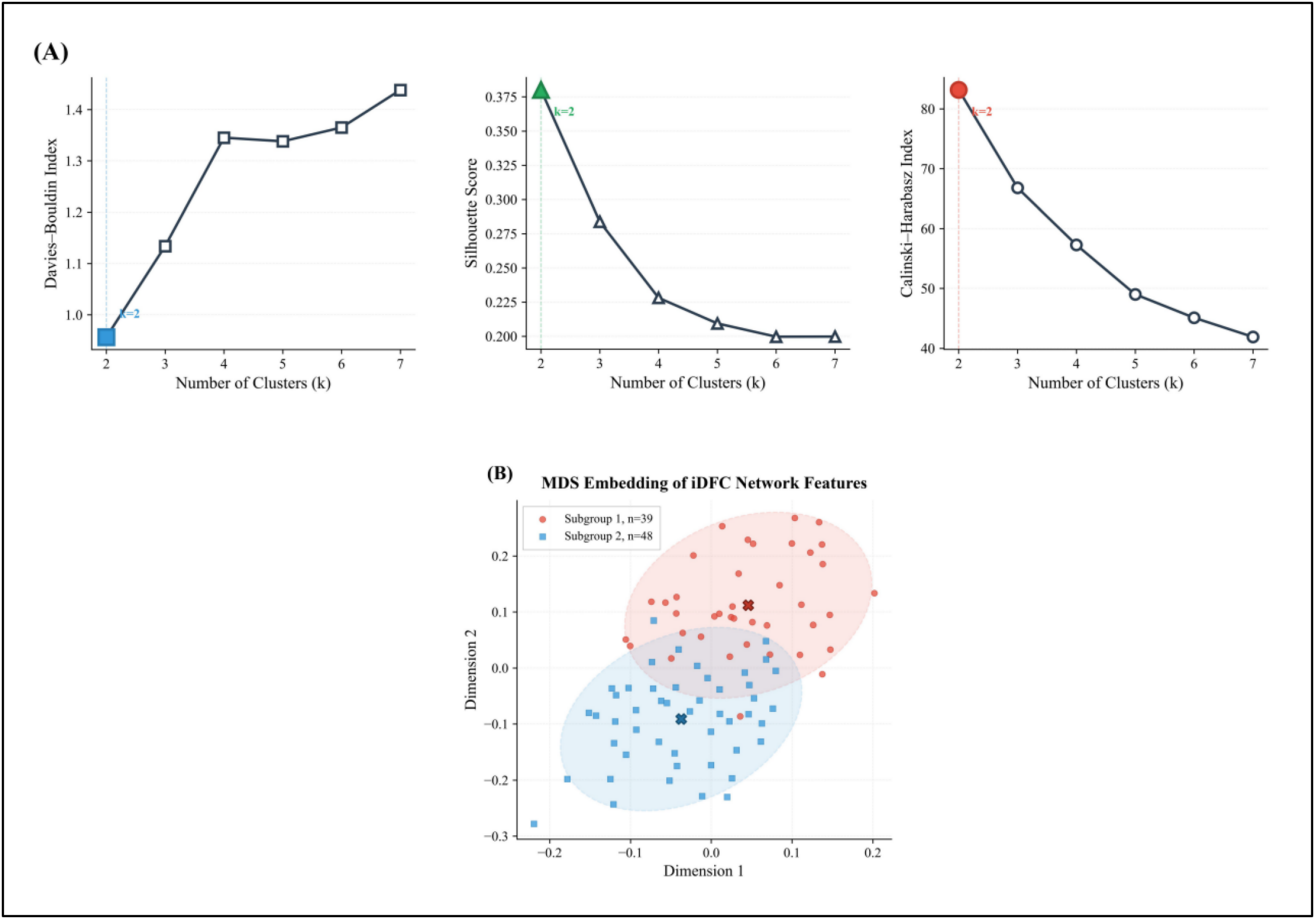
Clustering results of IGD subtypes based on network‐level IDFC. (A) Clustering evaluation metrics across different numbers of clusters (*k*). The optimal solution was achieved at *k* = 2, as indicated by the clustering evaluation metrics. (B) Visualization of the clustering results using *k*‐means based on network‐level IDFC features. A total of 87 patients with IGD were classified into two subtypes, including Subgroup 1 (*n* = 39) and Subgroup 2 (*n* = 48).

Based on network‐level IDFC features, *k*‐means clustering identified two IGD subgroups. Clustering evaluation indicated that the optimal solution was *k* = 2, with a Calinski–Harabasz index of 83.19, a Davies–Bouldin index of 0.956 and a silhouette score of 0.380 (Figure 2A). Under this solution, patients with IGD were divided into Subgroup 1 (*n* = 39) and Subgroup 2 (*n* = 48) (Figure 2B). Subsampling analysis demonstrated stable clustering performance, with a mean ARI of 0.8193 ± 0.0968 and a mean NMI of 0.7666 ± 0.1057 across 1000 iterations (Figure S1). Alternative clustering analyses showed relatively strong agreement for Ward hierarchical clustering (ARI = 0.7006 and NMI = 0.6704) and moderate agreement for Gaussian mixture modelling (ARI = 0.4536 and NMI = 0.3600) and signed‐FC sensitivity analysis (ARI = 0.4537 and NMI = 0.4771). In contrast, global‐FC‐regressed clustering showed low agreement with the primary solution (ARI = 0.1522 and NMI = 0.1263), indicating that global FC variation contributed substantially to the current subtype structure (Figure S2). As summarized in Table S7, the sex distribution did not differ significantly between Subgroups 1 and 2 (31/8 vs. 40/8 male/female, chi‐square = 0.212, *p* = 0.645). Subgroup 1 showed higher IAT scores than Subgroup 2 (69.46 ± 12.53 vs. 64.06 ± 9.81, *p* = 0.031) and higher HAMA scores (17.49 ± 8.16 vs. 12.58 ± 11.41, *p* = 0.022), whereas HAMD, Y‐BOCS and mania scores did not differ significantly between subgroups.

Exploratory within‐subgroup Spearman correlation analyses further examined associations between the 11 network‐level IDFC features and clinical scale scores (IAT/Young, HAMD, HAMA, Y‐BOCS and mania), with FDR correction across the 11 networks within each clinical scale and Fisher *r*‐to‐*z* tests comparing correlation coefficients between subgroups. The full exploratory correlation results are provided in Table S8.

### 3.2 Network Contingency Analysis of Edge‐Wise Functional Connectivity Alterations

**FIGURE 3:**
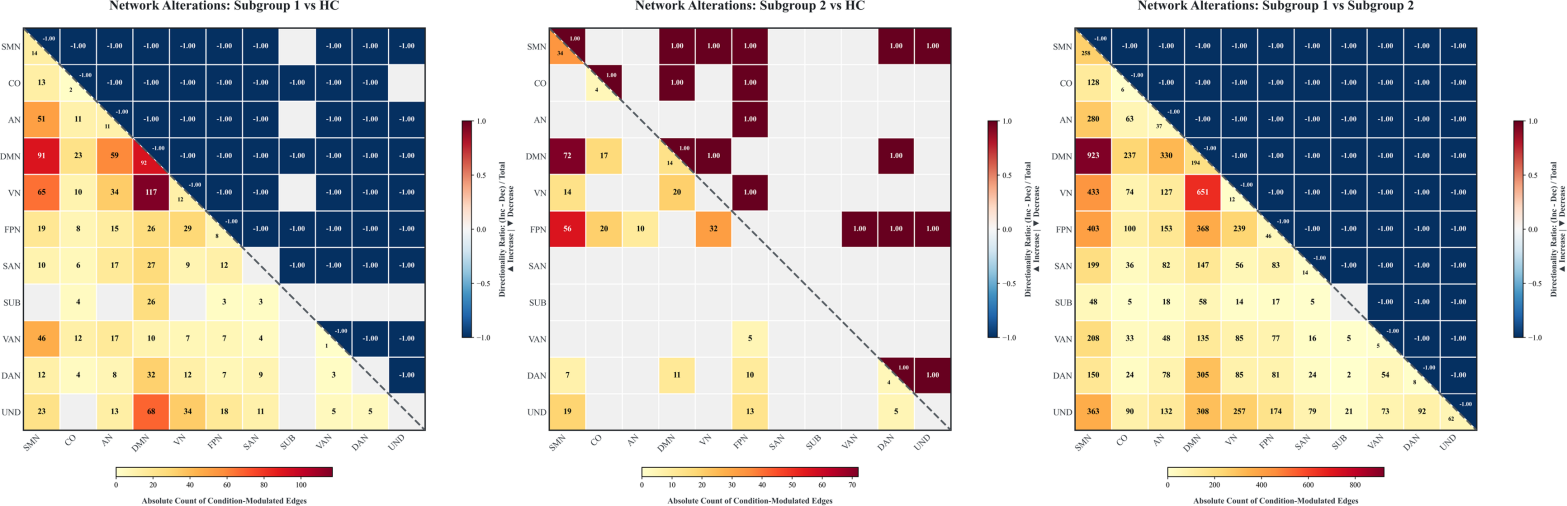
Network contingency analysis of functional connectivity alterations across large‐scale intrinsic connectivity networks (ICNs). The cross‐tabulation matrix illustrates the topological distribution of condition‐modulated edges identified at a primary threshold of *p* < 0.001 (uncorrected). The lower triangle represents the absolute number of altered edges within and between networks, reflecting the spatial extent of functional abnormalities. The upper triangle represents the directionality ratio, calculated as (number of increased edges − number of decreased edges) / total number of altered edges. A value of −1.00 (dark blue) indicates exclusively decreased connectivity, whereas +1.00 (dark red) indicates exclusively increased connectivity. Diagonal cells are split to display both the absolute count and the directionality ratio for within‐network connections. Only cells surviving FDR correction (*q* < 0.05) are colour‐coded; blank cells indicate no significant network‐level alterations. (A) Subgroup 1 versus HCs; (B) Subgroup 2 versus HCs; and (C) Subgroup 1 versus Subgroup 2. AN, auditory network; CO, cingulo‐opercular network; DAN, dorsal attention network; DMN, default mode network; FPN, frontoparietal network; SAN, salience network; SMN, sensorimotor network; SUB, subcortical network; UND, uncertain network; VAN, ventral attention network; VN, visual network.

NCA further characterized the network‐level distribution of altered FC edges across comparisons. In the comparison between Subgroup 1 and HCs, a total of 1207 suprathreshold edges were identified at the primary edge threshold of *p* < 0.001. All of these edges showed decreased FC in Subgroup 1 relative to HCs, resulting in a directionality ratio of −1.00 (Figure 3A). The altered edges were widely distributed across large‐scale intrinsic connectivity networks, indicating a broad hypoconnectivity pattern. Significant network‐pair alterations were observed in multiple within‐ and between‐network blocks after FDR correction, with prominent involvement of the DMN and its connections with other systems. In particular, decreased connectivity was evident around DMN‐related network pairs, supporting a DMN‐centred reduction in functional integration in Subgroup 1. In contrast, Subgroup 2 showed an increase‐dominant alteration pattern relative to HCs. A total of 437 suprathreshold edges were detected, including 435 increased edges and only 2 decreased edges, yielding a directionality ratio of 0.991 (Figure 3B). Compared with Subgroup 1, these alterations were less widespread and were concentrated in a smaller set of network pairs. These findings suggest that Subgroup 2 was characterized by relatively focal hyperconnectivity rather than global network‐wide disruption. The direct comparison between the two IGD subgroups showed the strongest edge‐wise difference. A total of 8918 suprathreshold edges were detected between Subgroups 1 and 2, and all of these edges showed lower FC in Subgroup 1 than in Subgroup 2 (directionality ratio = −1.00; Figure 3C). This result indicates a marked separation between the two subgroups along a hypoconnectivity to hyperconnectivity gradient. Significant network‐pair effects were observed across most large‐scale networks, suggesting that the subgroup difference was not restricted to a single isolated circuit but reflected a broad shift in functional connectivity organization. When all patients with IGD were combined into a single group, only 32 suprathreshold edges were identified relative to HCs, including 14 increased and 18 decreased edges (directionality ratio = −0.125). No comparably widespread network‐pair alteration pattern was observed in the combined IGD analysis. This contrast between the combined and subgroup‐specific analyses suggests that averaging across heterogeneous patients may obscure distinct and partially opposing FC alteration patterns. Overall, NCA indicated that Subgroup 1 was characterized by widespread hypoconnectivity, whereas Subgroup 2 showed a more focal increase‐dominant pattern. Additional NCA edge‐direction summaries and qFDR matrices are provided in Figures S4 and S5.

### 3.3 Group Differences in Global Functional Connectivity

Significant group differences in Global FC were observed among the three groups. HCs showed intermediate Global FC (0.2165 ± 0.0894, *n* = 56), Subgroup 1 showed the lowest Global FC (0.1609 ± 0.0301, *n* = 39) and Subgroup 2 showed the highest Global FC (0.2639 ± 0.0923, *n* = 48). The overall group effect was significant (ANOVA, *p* = 8.762 × 10–8). Post hoc comparisons with Bonferroni correction showed that Subgroup 1 had significantly lower Global FC than both HCs (corrected *p* = 1.494 × 10–4) and Subgroup 2 (corrected *p* = 2.801 × 10–9), whereas Subgroup 2 had significantly higher Global FC than HCs (corrected *p* = 0.0282) (Figure S3). These findings support a global hypoconnectivity to hyperconnectivity gradient across the IDFC‐defined IGD subgroups.

### 3.4 Prediction of IAT Scores Using Network‐Level IDFC Features

**FIGURE 4:**
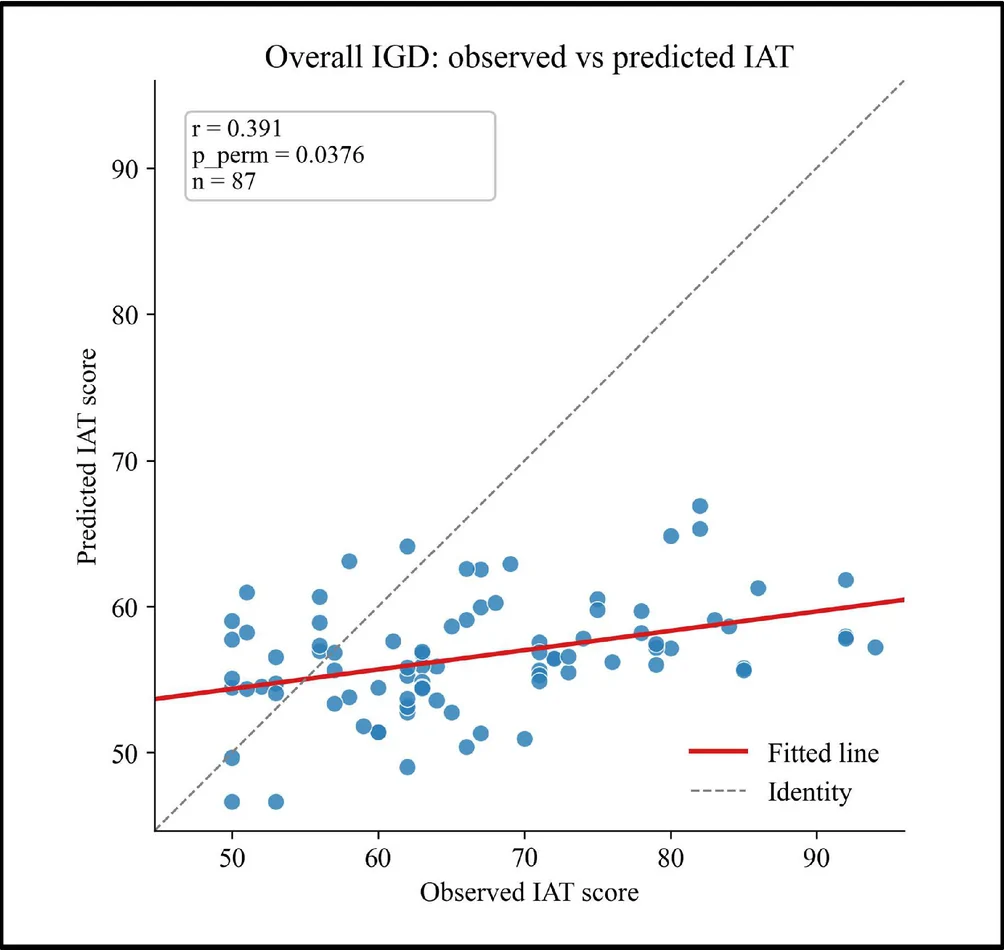
Prediction of IAT Scores Using Network‐Level IDFC Features in the Overall IGD Cohort Scatter plot showing the relationship between predicted and observed IAT scores based on network‐level IDFC features in the overall IGD cohort. Prediction performance was evaluated using LOOCV. The red line represents the fitted regression line, and the dashed line indicates the identity line (*y* = *x*). Pearson correlation coefficient (*r*) and one‐tailed permutation‐based *p* value (*p_perm*) are displayed in the panel.

SVR analysis showed that network‐level IDFC features predicted IAT scores across the overall IGD cohort. Predicted IAT scores were positively correlated with observed scores (*r* = 0.391, one‐tailed permutation *p* = 0.038; Figure 4). The model showed a mean absolute error of 11.61 and a root mean squared error of 14.40. To improve interpretability, feature weights were averaged across LOOCV iterations. The largest absolute weights were observed in the cingulo‐opercular (CO), dorsal attention (DAN), subcortical (SUB), uncertain (UND), sensory‐motor (SMN) and default mode (DMN) networks. The averaged feature‐weight profile is provided in the Supporting Information.

## 4 Discussion

A central point of this study is that heterogeneity in IGD should not be treated simply as residual variance around a case–control effect. When patients were characterized by network‐level interindividual deviation of functional connectivity (IDFC), two connectivity‐defined subgroups emerged. They were not minor variants of the same group‐average abnormality. Subgroup 1 showed lower Global FC, widespread hypoconnectivity, prominent DMN involvement and higher IAT scores. Subgroup 2 showed higher Global FC and a largely increase‐dominant pattern involving sensorimotor, frontoparietal, default‐mode and attention‐related systems. The analytic sequence is important here. IDFC features were used to identify subgroups; conventional FC, NCA and Global FC analyses were then used to describe what those subgroups looked like neurobiologically. This distinction reduces the risk of circular interpretation and makes the main finding more specific: IGD in this sample was better described by at least two macroscale connectivity configurations than by a single dysconnectivity profile.

### 4.1 IGD Heterogeneity as a Connectomic Problem

These findings help explain why previous FC studies of IGD have not always converged on the same pattern of abnormalities. Part of this inconsistency may come from genuine patient heterogeneity rather than methodological noise alone. Clinical studies have already shown that gaming disorder can be divided into symptom‐ or personality‐based subgroups [4, 33], and recent neuroimaging work has suggested that individualized brain measures can reveal latent subtypes in psychiatric and addictive disorders [10, 12, 13, 14, 15]. In the present study, the two subgroups were defined from IDFC patterns rather than from clinical scores. Even so, Subgroup 1 showed higher IAT scores and higher anxiety‐related scores than Subgroup 2. This finding is important because it links the brain‐based stratification to a clinically meaningful difference: The subgroup with the more widespread hypoconnectivity pattern also showed greater addiction severity and anxiety burden. In this sense, our results are consistent with previous clinical subtyping studies, but they add a connectomic basis for such heterogeneity. The stability analyses further suggest that the two‐cluster solution was not random, whereas the sensitivity analyses indicate that positive global FC variation contributed substantially to the low‐ versus high‐connectivity separation. Thus, in this dataset, global connectivity should not be dismissed automatically as a nuisance signal. It may be part of the phenotype that separates IGD subgroups, although this interpretation still needs replication and careful testing across preprocessing strategies.

### 4.2 Two Network Configurations, Not One Abnormality

Subgroup 1 had the more severe clinical profile and the more diffuse reduction in connectivity. At the primary edge threshold, all 1207 suprathreshold edges were decreased relative to HCs. The NCA results placed much of this effect around the DMN and its links with sensorimotor, visual, frontoparietal and underdetermined networks. This pattern is biologically plausible. The DMN supports self‐referential processing, internally directed cognition, affective appraisal and autobiographical functions [34, 35, 36], and DMN dysfunction has repeatedly been implicated in addictive disorders [37, 38]. Reduced DMN‐FPN coupling is also relevant because the frontoparietal control system helps coordinate internally directed thought with goal‐directed control [39, 40]. In practical terms, a DMN‐centred hypoconnected pattern may reflect poorer integration between self‐monitoring, control and motivational systems when gaming urges compete with longer term goals. That interpretation is consistent with the higher IAT scores in Subgroup 1, but the direction of effect cannot be inferred from these data. Hypoconnectivity could be a vulnerability marker, a consequence of persistent gaming or a state‐related correlate of symptom burden.

Subgroup 2 should not be described as the simple opposite of Subgroup 1. It showed higher Global FC than HCs and 435 of 437 suprathreshold altered edges were increased, but higher connectivity is not automatically healthier or compensatory. The affected systems, including sensorimotor, frontoparietal, default‐mode and attention‐related networks, point instead to a different resting‐state configuration. One possibility is that this subgroup has stronger coupling among systems repeatedly engaged during gaming: visuomotor readiness, attentional orienting, performance monitoring and flexible control [16, 28, 29, 41, 42]. Another possibility is reduced network segregation, where broad coupling makes the system less specialized or less flexible. Both interpretations are compatible with current network theory, in which abnormal integration can arise from either too little communication across control and association systems or too much coupling across systems that normally remain partly segregated [43, 44]. This is why the two subgroups are better framed as different network modes rather than as a severe versus mild version of the same abnormality.

### 4.3 Severity Is Still Dimensional

The prediction analysis adds a useful counterpoint to the categorical subgroup result. Network‐level IDFC features predicted IAT scores across the full IGD cohort, indicating that individualized deviations also carry dimensional information about symptom severity. The largest averaged absolute weights involved CO, DAN, SUB, UND, SMN and DMN features. This distribution argues against a single‐network account of addiction severity. It instead points to a broader set of deviations involving salience/cingulo‐opercular control, attention, subcortical circuitry, sensorimotor systems and default‐mode processes. The categorical and dimensional findings therefore address different levels of the same problem. The subgroups describe qualitatively different connectomic patterns; the SVR model shows that severity varies along distributed network deviations across patients. The prediction result should nevertheless be kept in proportion. Cross‐validation and permutation testing support an association within this cohort, but without external validation, the model is not a clinical biomarker [32, 45, 46].

### 4.4 Implications for Stratified IGD Research

These subgroup findings are not yet ready to guide treatment decisions, but they do suggest how future IGD intervention studies could be designed more precisely. For patients resembling Subgroup 1, the key question may be whether reduced DMN‐centred connectivity is related to poorer self‐monitoring, weaker cognitive control or greater difficulty disengaging from gaming‐related thoughts and urges. Cognitive‐behavioural approaches for this subgroup may therefore place greater emphasis on craving monitoring, cognitive restructuring, impulse‐control training and strategies for interrupting repetitive gaming‐related thoughts. In more severe cases, neuromodulation studies may also consider whether targeting prefrontal control regions can improve DMN‐control network coordination and reduce addiction severity [47, 48]. For patients resembling Subgroup 2, the clinical focus may be different. Because this subgroup showed elevated Global FC and stronger coupling among sensorimotor, attentional and control‐related systems, behavioural interventions may need to focus more on cue reactivity, habitual gaming routines, attentional disengagement from gaming cues and replacement of automatic gaming behaviours with alternative action patterns. For severe patients in this subgroup, TMS protocols might be designed to modulate frontoparietal or sensorimotor‐attentional circuits, rather than focusing only on DMN‐control dysregulation. These possibilities should be tested prospectively in longitudinal or treatment‐outcome studies, because resting‐state FC alone cannot determine whether either pattern is a vulnerability marker, a consequence of gaming or a state‐dependent feature.

### 4.5 Limitations

Several limitations need to be kept in view. First, the sample size was moderate for a study focused on interindividual variation, especially for Subgroup 1, and the single‐centre cross‐sectional design prevents conclusions about causality or subtype stability. Although the internal robustness analyses supported the primary clustering solution, internal robustness cannot substitute for independent external validation. Larger independent multisite datasets are needed to determine whether these patterns are stable traits, state‐dependent configurations or sample‐specific clusters. Second, residual confounding remains possible despite adjustment for age, sex, education and head motion. Developmental stage, gaming genre, daily gaming duration, medication exposure, socioeconomic background, sleep, cognition, pubertal indices and subthreshold affective or anxiety symptoms were not fully characterized. Third, *k*‐means clustering assumes relatively simple cluster geometry and can be sensitive to preprocessing. The supplementary analyses partly address this concern, but future studies should compare clustering methods, normative‐model approaches, signed‐FC pipelines and global‐signal‐related sensitivity analyses. Fourth, static resting‐state FC cannot capture temporal flexibility. Dynamic connectivity analyses may clarify whether the subgroups differ in dwell time, transition patterns or moment‐to‐moment network reconfiguration [49]. Fifth, the SVR model needs external validation before it can support translational claims. Finally, the small number of female participants limits conclusions about sex‐specific patterns, which is important given reported sex differences in IGD‐related neural responses [50].

## 5 Conclusion

In summary, the findings argue for a more stratified view of IGD connectomics. One subgroup showed a DMN‐centred hypoconnected pattern with lower Global FC and higher addiction severity. The other showed higher Global FC and an increase‐dominant pattern involving sensorimotor, frontoparietal, default‐mode and attention‐related systems. Network‐level IDFC also predicted IAT scores, suggesting that individualized connectivity deviations contain dimensional information about symptom burden. Taken together, these preliminary results suggest that IDFC‐based mapping may be useful for studying IGD heterogeneity, but they require replication in larger independent multisite cohorts, longitudinal follow‐up and treatment‐outcome studies before the subgroups can be used clinically.

## Author Contributions

**Yichen Guo:** conceptualization, methodology, formal analysis, data curation, visualization, writing – original draft. **Xinyu Wang:** methodology, investigation, writing – review and editing. **Wenjing Li:** investigation, data curation. **Guohao Lu:** investigation, data curation. **Yang Liu:** investigation, data curation. **Sha Zhou:** investigation, data curation. **Yan Lang:** resources, writing – review and editing. **Yong Zhang:** supervision, project administration, writing – review and editing, correspondence. **Weijian Wang:** conceptualization, funding acquisition, supervision, writing – review and editing, correspondence.

## Funding

This work was supported by the National Natural Science Foundation of China (No. 82471962), the Special Project for Scientific Research on Traditional Chinese Medicine in Henan Province in 2024 (No. 2024ZY3085), the Key Research Project Program for Higher Education Institutions in Henan Province in 2024 (No. 24A320059) and the Henan Provincial Medical Science and Technology Research Project Major Project Jointly Established by Provincial and Ministerial Authorities (No. SBGJ202101013).

## Ethics Statement

This study involving human participants was conducted in accordance with the ethical standards of the local medical ethics committee of the First Affiliated Hospital of Zhengzhou University. Written informed consent was obtained from all participants before their involvement in the study.

## Conflicts of Interest

The authors declare no conflicts of interest.

## Supporting information


**Table S1:** Power atlas network assignment used for network‐level IDFC and NCA.
**Table S2:** Clustering evaluation metrics across k = 2–7.
**Table S3:** Subsampling stability summary of the two‐cluster solution.
**Table S4:** Alternative clustering and sensitivity analyses.
**Table S5:** Edge‐direction summary of NCA across group comparisons.
**Table S6:** Significant network pairs identified by NCA.
**Table S7:** Sex and clinical scale comparisons between IGD subgroups.
**Table S8:** Exploratory within‐subgroup correlations between network‐level IDFC features and clinical scale scores.
**Figure S1:** Empirical distribution of clustering stability metrics across 1,000 subsampling iterations.
**Figure S2:** Agreement between the primary k‐means solution and alternative clustering/sensitivity analyses.
**Figure S3:** Positive global functional connectivity across HCs and IGD subgroups.
**Figure S4:** NCA edge‐direction summary across comparisons.
**Figure S5:** Complete NCA qFDR matrices for subgroup comparisons.

## Data Availability

The data that support the findings of this study are available from the corresponding author upon reasonable request.
