# Supplementary material for: Subtyping Internet Gaming Disorder Based on Interindividual Deviation of Functional Connectivity: A Network Contingency Analysis
Source: Addict Biol. 2026 Sep 10;31(9):e70184. doi: 10.1111/adb.70184 (PMC13559469; doi:10.1111/adb.70184)
Supplement: Supplementary file 1 [file ADB-31-e70184-s001.docx]

**Supplementary Material**

# S1. IDFC construction and clustering preprocessing

Functional connectivity matrices were constructed using the Power 264-region atlas and Fisher z-transformed before downstream analyses. For the primary positive-only FC analysis, negative FC values were set to zero. For each IGD participant, ROI-level individualized functional connectivity distance (IDFC) was computed using cosine distance between the participant-specific ROI FC profile and the corresponding HC reference profiles. ROI-level IDFC values were then averaged within the 11 Power atlas network assignments to obtain network-level IDFC features. Prior to clustering, age, sex, mean framewise displacement (mean FD), and education were regressed from the network-level IDFC features, and the residualized features were standardized. The primary clustering solution used k-means with k = 2.

# S2. Clustering stability and sensitivity analyses

Stability of the two-cluster solution was assessed using 1,000 iterations of 80% subsampling. In each iteration, the full preprocessing sequence was repeated, including covariate regression, standardization, and k-means clustering. Agreement between subsampling-derived labels and the primary full-sample labels was quantified using the adjusted Rand index (ARI) and normalized mutual information (NMI). Additional sensitivity analyses included Ward hierarchical clustering, Gaussian mixture modeling, clustering based on signed FC, and clustering after regression of individual positive global FC. These analyses were used to assess whether the primary solution reflected a robust low-/high-connectivity biotype structure and to quantify the contribution of global FC variation.

# S3. Edge-wise FC an**alysis with network contingency anal**ysis

For NCA, edge-wise general linear models were fitted across the 34,716 unique upper-triangular Power264 edges. The primary suprathreshold edge definition was p < 0.001 uncorrected at the edge level. For each network pair, the observed number of suprathreshold edges was counted. Statistical inference at the network-pair level used Freedman-Lane residual permutation with 10,000 permutations, followed by FDR correction across network pairs. Total, increased, and decreased edge counts were summarized separately. Directionality was quantified as (number of increased edges - number of decreased edges) / total number of suprathreshold edges. NCA should be interpreted as a network-level distribution analysis of altered edges rather than as conventional graph-theoretical topology.

**S4. Supplementary SVR feature-weight profile**

For the overall IGD cohort, feature weights from the LinearSVR model were averaged across LOOCV folds to summarize the relative contribution of the 11 network-level IDFC features. These weights are provided as Supplementary Figure S6.

# Supplementary Tables

**Table S1. Power atlas network assignment used for network-level IDFC and NCA.**

| **Network abbreviation** | **Network name** | **Number of ROIs** |
| --- | --- | --- |
| SMN | sensorimotor network | 35 |
| CO | cingulo-opercular network | 14 |
| AN | auditory network | 13 |
| DMN | default mode network | 58 |
| VN | visual network | 31 |
| FPN | frontoparietal network | 25 |
| SAN | salience network | 18 |
| SUB | subcortical network | 13 |
| VAN | ventral attention network | 9 |
| DAN | dorsal attention network | 11 |
| UND | undefined/unassigned network | 37 |

*Note.* ROI counts were computed from the Power264 network mapping file used in the current IDFC/NCA workflow. Network abbreviations are kept in the fixed order used for all NCA matrices.

**Table S2. Clustering evaluation metrics across k = 2–7.**

| **k** | **Calinski-Harabasz index** | **Davies-Bouldin index** | **Silhouette score** | **Inertia** |
| --- | --- | --- | --- | --- |
| 2 | 83.19 | 0.9565 | 0.3802 | 483.7 |
| 3 | 66.8 | 1.134 | 0.2838 | 369.4 |
| 4 | 57.28 | 1.345 | 0.2281 | 311.7 |
| 5 | 49.01 | 1.338 | 0.2094 | 282.2 |
| 6 | 45.1 | 1.365 | 0.1997 | 252.9 |
| 7 | 41.9 | 1.438 | 0.1998 | 231 |

*Note.* These values are read from the current cluster_evaluation.csv file. For k = 2, the current values are Calinski-Harabasz = 83.1854, Davies-Bouldin = 0.9565, and silhouette = 0.3802; these values should be used consistently in the manuscript and supplement.

**Table S3. Subsampling stability summary of the two-cluster solution.**

| **Metric** | **Mean** | **SD** | **Median** | **Minimum** | **Maximum** | **5th percentile** | **25th percentile** | **75th percentile** | **95th percentile** | **≥0.70 proportion** | **≥0.80 proportion** |
| --- | --- | --- | --- | --- | --- | --- | --- | --- | --- | --- | --- |
| ARI | 0.8193 | 0.09677 | 0.8335 | 0.4246 | 1 | 0.6815 | 0.7309 | 0.8874 | 1 | 89.0% | 53.5% |
| NMI | 0.7666 | 0.1057 | 0.7461 | 0.4635 | 1 | 0.605 | 0.6911 | 0.8388 | 1 | 66.6% | 34.8% |

*Note.* Stability statistics were computed from the 1,000-iteration robustness_subsampling_ari.csv file, not from simulated values. ARI = adjusted Rand index; NMI = normalized mutual information.

**Table S4. Alternative clustering and sensitivity analyses.**

| **Analysis** | **Cluster sizes** | **ARI vs primary k-means** | **NMI vs primary k-means** | **Interpretation** |
| --- | --- | --- | --- | --- |
| Ward hierarchical | 46 / 41 | 0.7006 | 0.6704 | Relatively strong agreement with the primary solution |
| Gaussian mixture model | 39 / 48 | 0.4536 | 0.36 | Moderate agreement with the primary solution |
| Signed-FC sensitivity | 25 / 62 | 0.4537 | 0.4771 | Moderate agreement, indicating partial dependence on positive-only FC preprocessing |
| Global-FC-regressed clustering | 62 / 25 | 0.1522 | 0.1263 | Low agreement, indicating a substantial contribution of global FC variation |

*Note.* The global-FC-regressed analysis produced low agreement with the primary clustering solution, indicating that individual positive global FC variation substantially contributes to the current low-/high-connectivity biotype structure. This result should be reported transparently and should not be described as evidence that clustering is independent of global FC.

**Table S5. Edge-direction summary of NCA across group comparisons.**

| **Comparison** | **n group A** | **n group B** | **Total p<0.001 edges** | **Increased edges** | **Decreased edges** | **Directionality ratio** | **Permutations** | **Permutation method** |
| --- | --- | --- | --- | --- | --- | --- | --- | --- |
| Combined_IGD_vs_HC | 87 | 56 | 32 | 14 | 18 | -0.125 | 10000 | freedman_lane |
| Subgroup1_vs_HC | 39 | 56 | 1207 | 0 | 1207 | -1 | 10000 | freedman_lane |
| Subgroup2_vs_HC | 48 | 56 | 437 | 435 | 2 | 0.9908 | 10000 | freedman_lane |
| Subgroup1_vs_Subgroup2 | 39 | 48 | 8918 | 0 | 8918 | -1 | 10000 | freedman_lane |

*Note.* Increased and decreased edges are defined relative to group A in each comparison. The directionality ratio is (increased - decreased) / total suprathreshold edges. Edge threshold was p < 0.001 uncorrected; network-pair inference used 10,000 Freedman-Lane permutations.

**Table S6. Significant network pairs identified by NCA.**

| **Comparison** | **Direction type** | **Network i** | **Network j** | **Observed edges** | **Permutation p** | **FDR q** |
| --- | --- | --- | --- | --- | --- | --- |
| Subgroup1_vs_HC | DECREASE | SMN | AN | 51 | 9.999e-05 | 0.0004714 |
| Subgroup1_vs_HC | DECREASE | SMN | DMN | 91 | 9.999e-05 | 0.0004714 |
| Subgroup1_vs_HC | DECREASE | SMN | VN | 65 | 9.999e-05 | 0.0004714 |
| Subgroup1_vs_HC | DECREASE | SMN | VAN | 46 | 9.999e-05 | 0.0004714 |
| Subgroup1_vs_HC | DECREASE | CO | VAN | 12 | 9.999e-05 | 0.0004714 |
| Subgroup1_vs_HC | DECREASE | AN | AN | 11 | 9.999e-05 | 0.0004714 |
| Subgroup1_vs_HC | DECREASE | AN | DMN | 59 | 9.999e-05 | 0.0004714 |
| Subgroup1_vs_HC | DECREASE | AN | VN | 34 | 9.999e-05 | 0.0004714 |
| Subgroup1_vs_HC | DECREASE | AN | VAN | 17 | 9.999e-05 | 0.0004714 |
| Subgroup1_vs_HC | DECREASE | DMN | DMN | 92 | 9.999e-05 | 0.0004714 |
| Subgroup1_vs_HC | DECREASE | DMN | VN | 117 | 9.999e-05 | 0.0004714 |
| Subgroup1_vs_HC | DECREASE | DMN | SAN | 27 | 9.999e-05 | 0.0004714 |
| Subgroup1_vs_HC | DECREASE | DMN | SUB | 26 | 9.999e-05 | 0.0004714 |
| Subgroup1_vs_HC | DECREASE | DMN | UND | 68 | 9.999e-05 | 0.0004714 |
| Subgroup1_vs_HC | DECREASE | AN | SAN | 17 | 0.0002 | 0.0006947 |
| Subgroup1_vs_HC | DECREASE | AN | DAN | 8 | 0.0002 | 0.0006947 |
| Subgroup1_vs_HC | DECREASE | DMN | DAN | 32 | 0.0002 | 0.0006947 |
| Subgroup1_vs_HC | DECREASE | VN | UND | 34 | 0.0002 | 0.0006947 |
| Subgroup1_vs_HC | DECREASE | SAN | DAN | 9 | 0.0002 | 0.0006947 |
| Subgroup1_vs_HC | DECREASE | CO | DMN | 23 | 0.0003 | 0.0008608 |
| Subgroup1_vs_HC | DECREASE | AN | UND | 13 | 0.0003 | 0.0008608 |
| Subgroup1_vs_HC | DECREASE | VN | DAN | 12 | 0.0003 | 0.0008608 |
| Subgroup1_vs_HC | DECREASE | FPN | SAN | 12 | 0.0003 | 0.0008608 |
| Subgroup1_vs_HC | DECREASE | CO | AN | 11 | 0.0004 | 0.001056 |
| Subgroup1_vs_HC | DECREASE | VN | FPN | 29 | 0.0004 | 0.001056 |
| Subgroup1_vs_HC | DECREASE | AN | FPN | 15 | 0.0005999 | 0.001467 |
| Subgroup1_vs_HC | DECREASE | FPN | UND | 18 | 0.0005999 | 0.001467 |
| Subgroup1_vs_HC | DECREASE | SMN | UND | 23 | 0.0008999 | 0.001856 |
| Subgroup1_vs_HC | DECREASE | DMN | FPN | 26 | 0.0008999 | 0.001856 |
| Subgroup1_vs_HC | DECREASE | VN | VN | 12 | 0.0008999 | 0.001856 |
| Subgroup1_vs_HC | DECREASE | FPN | VAN | 7 | 0.0008999 | 0.001856 |
| Subgroup1_vs_HC | DECREASE | SAN | UND | 11 | 0.0007999 | 0.001856 |
| Subgroup1_vs_HC | DECREASE | SMN | SMN | 14 | 0.0011 | 0.002016 |
| Subgroup1_vs_HC | DECREASE | SMN | CO | 13 | 0.0011 | 0.002016 |
| Subgroup1_vs_HC | DECREASE | SMN | DAN | 12 | 0.0011 | 0.002016 |
| Subgroup1_vs_HC | DECREASE | FPN | DAN | 7 | 0.0011 | 0.002016 |
| Subgroup1_vs_HC | DECREASE | DMN | VAN | 10 | 0.0012 | 0.002084 |
| Subgroup1_vs_HC | DECREASE | FPN | FPN | 8 | 0.0012 | 0.002084 |
| Subgroup1_vs_HC | DECREASE | CO | VN | 10 | 0.0013 | 0.002145 |
| Subgroup1_vs_HC | DECREASE | CO | DAN | 4 | 0.0013 | 0.002145 |
| Subgroup1_vs_HC | DECREASE | VN | VAN | 7 | 0.0016 | 0.002575 |
| Subgroup1_vs_HC | DECREASE | CO | SAN | 6 | 0.0017 | 0.002671 |
| Subgroup1_vs_HC | DECREASE | SMN | FPN | 19 | 0.0018 | 0.0027 |
| Subgroup1_vs_HC | DECREASE | CO | SUB | 4 | 0.0018 | 0.0027 |
| Subgroup1_vs_HC | DECREASE | CO | FPN | 8 | 0.002 | 0.002869 |
| Subgroup1_vs_HC | DECREASE | SAN | VAN | 4 | 0.002 | 0.002869 |
| Subgroup1_vs_HC | DECREASE | VAN | DAN | 3 | 0.0022 | 0.003025 |
| Subgroup1_vs_HC | DECREASE | VAN | UND | 5 | 0.0022 | 0.003025 |
| Subgroup1_vs_HC | DECREASE | VN | SAN | 9 | 0.0025 | 0.003367 |
| Subgroup1_vs_HC | DECREASE | SMN | SAN | 10 | 0.004 | 0.005279 |
| Subgroup1_vs_HC | DECREASE | DAN | UND | 5 | 0.0041 | 0.005305 |
| Subgroup1_vs_HC | DECREASE | SAN | SUB | 3 | 0.005899 | 0.007488 |
| Subgroup1_vs_HC | DECREASE | CO | CO | 2 | 0.006299 | 0.007844 |
| Subgroup1_vs_HC | DECREASE | FPN | SUB | 3 | 0.0102 | 0.01247 |
| Subgroup1_vs_HC | DECREASE | VAN | VAN | 1 | 0.0157 | 0.01884 |
| Subgroup1_vs_HC | DECREASE | SUB | SUB | 1 | 0.0252 | 0.0297 |
| Subgroup1_vs_HC | DECREASE | SMN | SUB | 2 | 0.0372 | 0.04307 |
| Subgroup1_vs_HC | DECREASE | VN | SUB | 2 | 0.0381 | 0.04335 |
| Subgroup1_vs_HC | TOTAL | SMN | AN | 51 | 9.999e-05 | 0.0009428 |
| Subgroup1_vs_HC | TOTAL | CO | VAN | 12 | 9.999e-05 | 0.0009428 |

*Note.* This table lists the first 60 significant network-pair rows sorted by comparison, direction type, and FDR q value. The complete table contains 285 significant rows with qFDR < 0.05 and is provided as Supplementary Data: all_significant_network_pairs_summary.csv.

Table S7. Sex and clinical scale comparisons between IGD subgroups.

Note. Values are mean ± SD for clinical scales unless otherwise indicated. The t/χ² value column reports Welch’s t statistics for clinical scale comparisons and the chi-square statistic for sex distribution. Sex distribution was compared using a chi-square test, and clinical scale scores were compared using Welch two-sample t-tests because subgroup variances and available sample sizes differed across clinical scales. IAT, Internet Addiction Test; HAMD, Hamilton Depression Scale; HAMA, Hamilton Anxiety Scale; Y-BOCS, Yale-Brown Obsessive Compulsive Scale.

| **Measure** | **Subgroup 1** | **Subgroup 2** | t/χ² value | **p value** |
| --- | --- | --- | --- | --- |
| IAT score | 69.46 ± 12.53 (n=39) | 64.06 ± 9.81 (n=48) | 2.199 | 0.031 |
| HAMD score | 21.30 ± 11.47 (n=33) | 22.05 ± 15.20 (n=44) | -0.244 | 0.808 |
| HAMA score | 17.49 ± 8.16 (n=39) | 12.58 ± 11.41 (n=48) | 2.333 | 0.022 |
| Y-BOCS score | 10.78 ± 8.28 (n=32) | 10.46 ± 8.65 (n=39) | 0.159 | 0.874 |
| Mania score | 8.41 ± 7.73 (n=29) | 8.89 ± 7.05 (n=38) | -0.262 | 0.794 |
| Sex, male/female, n | 31/8 | 40/8 | chi-square = 0.212 | 0.645 |

Table S8. Exploratory within-subgroup correlations between network-level IDFC features and clinical scale scores.

| Measure | Network | Subgroup 1 rho (qFDR) | Subgroup 2 rho (qFDR) | Fisher z | Between-subgroup p | Between-subgroup qFDR |
| --- | --- | --- | --- | --- | --- | --- |
| IAT | SMN | 0.276 (0.123) | -0.086 (0.815) | 1.652 | 0.099 | 0.217 |
| IAT | CO | 0.347 (0.056) | 0.246 (0.336) | 0.493 | 0.622 | 0.622 |
| IAT | AN | 0.436 (0.014) | 0.124 (0.815) | 1.535 | 0.125 | 0.229 |
| IAT | DMN | 0.243 (0.151) | 0.017 (0.991) | 1.032 | 0.302 | 0.458 |
| IAT | VN | 0.323 (0.070) | -0.208 (0.431) | 2.442 | 0.015 | 0.054 |
| IAT | FPN | 0.472 (0.009) | -0.002 (0.991) | 2.301 | 0.021 | 0.059 |
| IAT | SAN | 0.202 (0.216) | 0.359 (0.068) | -0.760 | 0.447 | 0.492 |
| IAT | SUB | 0.431 (0.014) | 0.586 (1.35e-04) | -0.941 | 0.347 | 0.458 |
| IAT | VAN | 0.257 (0.140) | 0.064 (0.815) | 0.888 | 0.375 | 0.458 |
| IAT | DAN | 0.495 (0.007) | -0.085 (0.815) | 2.809 | 0.005 | 0.034 |
| IAT | UND | 0.594 (7.45e-04) | 0.072 (0.815) | 2.736 | 0.006 | 0.034 |
| HAMD | SMN | 0.118 (0.989) | -0.004 (0.982) | 0.510 | 0.610 | 0.913 |
| HAMD | CO | 0.099 (0.989) | -0.194 (0.982) | 1.232 | 0.218 | 0.913 |
| HAMD | AN | 0.049 (0.989) | -0.214 (0.982) | 1.110 | 0.267 | 0.913 |
| HAMD | DMN | -0.012 (0.989) | -0.052 (0.982) | 0.167 | 0.868 | 0.954 |
| HAMD | VN | -0.106 (0.989) | 0.034 (0.982) | -0.582 | 0.560 | 0.913 |
| HAMD | FPN | 0.065 (0.989) | 0.008 (0.982) | 0.237 | 0.813 | 0.954 |
| HAMD | SAN | 0.003 (0.989) | -0.101 (0.982) | 0.434 | 0.664 | 0.913 |
| HAMD | SUB | -0.143 (0.989) | 0.047 (0.982) | -0.796 | 0.426 | 0.913 |
| HAMD | VAN | 0.046 (0.989) | -0.121 (0.982) | 0.698 | 0.485 | 0.913 |
| HAMD | DAN | -0.016 (0.989) | -0.010 (0.982) | -0.026 | 0.979 | 0.979 |
| HAMD | UND | -0.132 (0.989) | 0.164 (0.982) | -1.244 | 0.213 | 0.913 |
| HAMA | SMN | 0.338 (0.129) | 0.226 (0.273) | 0.547 | 0.585 | 0.805 |
| HAMA | CO | 0.525 (0.007) | 0.351 (0.160) | 0.972 | 0.331 | 0.805 |
| HAMA | AN | 0.280 (0.186) | -0.002 (0.988) | 1.295 | 0.195 | 0.805 |
| HAMA | DMN | -0.100 (0.876) | 0.286 (0.267) | -1.768 | 0.077 | 0.805 |
| HAMA | VN | -0.095 (0.876) | 0.069 (0.776) | -0.738 | 0.460 | 0.805 |
| HAMA | FPN | 0.059 (0.876) | 0.122 (0.640) | -0.288 | 0.773 | 0.826 |
| HAMA | SAN | 0.442 (0.026) | 0.211 (0.273) | 1.165 | 0.244 | 0.805 |
| HAMA | SUB | 0.294 (0.186) | 0.249 (0.273) | 0.220 | 0.826 | 0.826 |
| HAMA | VAN | -0.016 (0.924) | 0.093 (0.726) | -0.489 | 0.625 | 0.805 |
| HAMA | DAN | -0.043 (0.876) | 0.056 (0.776) | -0.441 | 0.659 | 0.805 |
| HAMA | UND | 0.047 (0.876) | 0.223 (0.273) | -0.801 | 0.423 | 0.805 |
| Y-BOCS | SMN | 0.056 (0.929) | -0.111 (0.967) | 0.672 | 0.502 | 0.731 |
| Y-BOCS | CO | 0.103 (0.929) | -0.131 (0.967) | 0.943 | 0.346 | 0.731 |
| Y-BOCS | AN | 0.031 (0.932) | -0.075 (0.967) | 0.427 | 0.670 | 0.818 |
| Y-BOCS | DMN | 0.170 (0.929) | -0.063 (0.967) | 0.942 | 0.346 | 0.731 |
| Y-BOCS | VN | 0.065 (0.929) | -0.096 (0.967) | 0.647 | 0.518 | 0.731 |
| Y-BOCS | FPN | 0.229 (0.929) | 0.005 (0.976) | 0.916 | 0.359 | 0.731 |
| Y-BOCS | SAN | 0.016 (0.932) | 0.012 (0.976) | 0.015 | 0.988 | 0.988 |
| Y-BOCS | SUB | -0.078 (0.929) | 0.078 (0.967) | -0.625 | 0.532 | 0.731 |
| Y-BOCS | VAN | 0.218 (0.929) | -0.070 (0.967) | 1.168 | 0.243 | 0.731 |
| Y-BOCS | DAN | 0.191 (0.929) | -0.019 (0.976) | 0.850 | 0.395 | 0.731 |
| Y-BOCS | UND | 0.084 (0.929) | 0.076 (0.967) | 0.033 | 0.974 | 0.988 |
| Mania | SMN | 0.037 (0.849) | 0.148 (0.717) | -0.433 | 0.665 | 0.914 |
| Mania | CO | 0.103 (0.849) | -0.122 (0.717) | 0.873 | 0.383 | 0.914 |
| Mania | AN | -0.114 (0.849) | -0.066 (0.761) | -0.184 | 0.854 | 0.917 |
| Mania | DMN | -0.133 (0.849) | 0.029 (0.862) | -0.630 | 0.528 | 0.914 |
| Mania | VN | -0.064 (0.849) | 0.133 (0.717) | -0.764 | 0.445 | 0.914 |
| Mania | FPN | 0.304 (0.849) | 0.328 (0.488) | -0.104 | 0.917 | 0.917 |
| Mania | SAN | 0.090 (0.849) | 0.230 (0.606) | -0.556 | 0.578 | 0.914 |
| Mania | SUB | 0.048 (0.849) | -0.091 (0.717) | 0.538 | 0.591 | 0.914 |
| Mania | VAN | -0.250 (0.849) | 0.134 (0.717) | -1.505 | 0.132 | 0.914 |
| Mania | DAN | 0.061 (0.849) | 0.107 (0.717) | -0.177 | 0.859 | 0.917 |
| Mania | UND | 0.052 (0.849) | 0.266 (0.584) | -0.854 | 0.393 | 0.914 |

Note. Values are Spearman rho, with qFDR corrected across the 11 network-level IDFC features within each clinical scale and subgroup. Fisher z and p values compare subgroup-specific correlation coefficients; between-subgroup qFDR values were corrected across the 11 networks within each clinical scale. The full exploratory correlation results are shown in this table, and machine-readable long-format results are provided in the accompanying CSV files: subgroup_idfc_clinical_spearman_long.csv and subgroup_idfc_clinical_spearman_fisher_full.csv. IDFC, inter-individual deviation of functional connectivity; FDR, false discovery rate.

# Supplementary Figures

**Figure S1. Empirical distribution of clustering stability metrics across 1,000 subsampling iterations.**

A, ARI distribution. B, NMI distribution. Vertical solid lines indicate the empirical mean, and dashed lines indicate the 5th and 95th percentiles. Values are computed from the observed robustness_subsampling_ari.csv file.

**Figure S2. Agreement between the primary k-means solution and alternative clustering/sensitivity analyses.**

Bars show ARI and NMI relative to the primary positive-only IDFC k-means solution. Global-FC-regressed clustering showed low agreement, supporting the interpretation that the primary subgroups are strongly aligned with a global FC gradient.

**Figure S3. Positive global functional connectivity across HCs and IGD subgroups.**

Violin, box, and jittered point plots show individual positive Global FC values in HCs, Subgroup 1, and Subgroup 2. Brackets indicate Bonferroni-corrected post hoc comparisons (* p < 0.05, *** p < 0.001). Subgroup 1 showed significantly lower Global FC than both HCs and Subgroup 2, whereas Subgroup 2 showed significantly higher Global FC than HCs. This pattern supports a global hypo- to hyperconnectivity gradient across the IDFC-defined IGD subgroups.

**Figure S4. NCA edge-direction summary across comparisons.**

A, total numbers of suprathreshold increased and decreased edges at p < 0.001. The y-axis uses a symmetric log scale to display both small and large edge counts. B, directionality ratio for each comparison. Positive values indicate increase-dominant alterations, whereas negative values indicate decrease-dominant alterations.

**Figure S5. Complete NCA qFDR matrices for subgroup comparisons.**

Matrices show -log10(qFDR) for TOTAL NCA inference across network pairs. Only qFDR < 0.05 network-pair blocks are colored. The color scale uses the manuscript heatmap style while preserving the supplementary qFDR-matrix result: light yellow denotes lower -log10(qFDR) values and dark red denotes higher -log10(qFDR) values. Matrix ordering follows the fixed Power264 network order: SMN, CO, AN, DMN, VN, FPN, SAN, SUB, VAN, DAN, UND.
